# Isoindolines and Isoindoline-1,3-diones as Nonpeptide ACE Inhibitors: An *In Silico* and *In Vitro* Modeling Approach

**DOI:** 10.1021/acsmedchemlett.5c00507

**Published:** 2026-02-09

**Authors:** Jessica E. Rodríguez, Jesús A. Lagos-Cruz, Rafael Villalobos-Molina, Roberto I. Cuevas-Hernández, Itzell A. Gallardo-Ortíz, Erik Andrade-Jorge

**Affiliations:** † Área de Química Computacional y Modelado Molecular, Bioquímica clínica, Carrera de Químico Farmacéutico Biólogo, Facultad de Estudios Superiores Zaragoza, 7180 Universidad Nacional Autónoma de México. Av. Guelatao con Av. Exploradores, Ejército de Oriente, Iztapalapa, 09320 Ciudad de México, México; ‡ Laboratorio de Investigación en Biomedicina y Toxicología, Sección de Estudios de Posgrado e Investigación. Escuela Superior de Medicina del Instituto Politécnico Nacional, Plan de San Luis y Díaz Mirón s/n Casco de Santo Tomás, 11340 Mexico City, México; § Unidad de Investigación en Biomedicina y Carrera de Enfermería, Facultad de Estudios Superiores-Iztacala, 7180 Universidad Nacional Autónoma de México. Av. de los Barrios 1, Los Reyes Iztacala, Tlalnepantla 54090, Estado de México, México

**Keywords:** Hypertension, ACE inhibitor, Enzyme kinetics, Lethal dose 50, Molecular docking

## Abstract

Hypertension, a major cardiovascular risk factor, is often treated with peptide-derived angiotensin-converting enzyme inhibitors (ACEi), which can have several side effects. This study examined a new alternative: isoindoline and isoindoline-1,3-dione derivatives as nonpeptide ACE inhibitors. The synthesis and testing of these compounds involved both *in silico* molecular docking studies and optimized *in vitro* inhibitory kinetic assays, along with acute toxicity tests in mice. isoindoline-1,3-dione, D-05, demonstrated the strongest ACE inhibition *in vitro* (IC_50_ = 416.4 μM) and effectively bound to the enzyme’s catalytic active site *in silico*. Additionally, isoindoline-1,3-diones showed lower toxicity in mice (LD_50_ > 1600 mg/kg) compared to isoindolines (LD_50_ < 1000 mg/kg). This reduced toxicity is attributed to the presence of fewer reactive secondary metabolites. These promising results highlight the potential of isoindoline-1,3-diones as innovative nonpeptide ACE inhibitors and support further *in vivo* studies to verify their antihypertensive effects.

## Graphical abstract

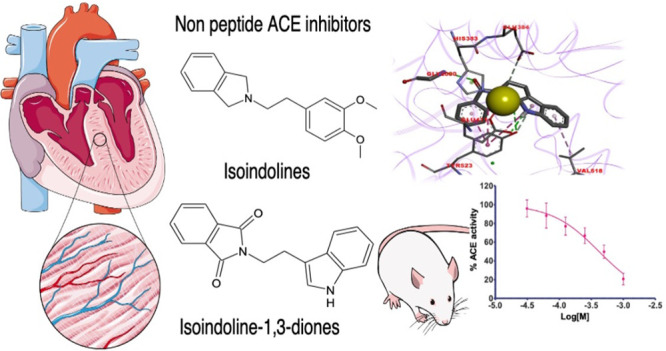

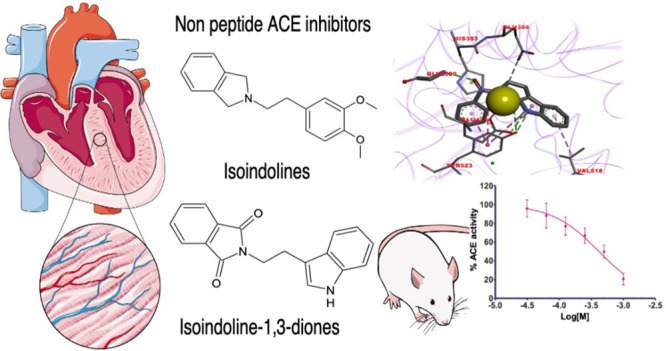

Hypertension (HTN), defined by consistently high systolic and diastolic blood pressure, is the leading global risk factor for coronary heart disease, heart failure, aortic and peripheral vascular disease, stroke, chronic kidney disease, among other complications.^1,2^ Estimates suggest over 1.3 billion adults worldwide suffer from high blood pressure.^3^ The main approach to tackling this global health issue is preventing HTN to avoid premature cardiovascular disease.^4^ The renin-angiotensin system (RAS) plays a central role in regulating blood pressure, making it a key biological target for treating HTN and other cardiovascular conditions. A crucial component of the RAS is angiotensin-converting enzyme (ACE, EC 3.4.15.1), a zinc dipeptidyl carboxypeptidase and a prime target for inhibitors.^5,6^ ACE converts angiotensin I (a decapeptide) into angiotensin II (an octapeptide) and hydrolyzes bradykinin, a powerful vasodilator.^7^ Angiotensin II acts as a potent vasoconstrictor while simultaneously deactivating bradykinin. Therefore, ACE’s activity, by excessively degrading bradykinin and angiotensin I and converting the latter into angiotensin II, is known to raise blood pressure, ultimately leading to HTN.^8^ Based on these insights, reducing bradykinin degradation and angiotensin II levels by inhibiting ACE has been the most effective strategy for preventing and treating HTN.^9^

In this context, although synthetic ACE inhibitors (ACEi), such as captopril and lisinopril, were developed in the 1970s, 13 family members are now widely used for the treatment of HTN.^10^ However, these approved ACEi come with a range of well-known side effects, including allergic reactions, reduced renal function, coughing, taste disorders, rash, among others.^11,12^ This is due to ACE’s influence, which extends beyond blood pressure regulation and impacts both the immune and central nervous systems.^13,14^ Furthermore, it has been reported that peptide-origin ACEi containing nitrogen-based heterocycles in their structure, such as enalapril, ramipril, and lisinopril, can cause angioedema, a serious side effect linked to their chemical structure. ACEi can cause angioedema by increasing bradykinin release. This action, along with the accumulation of several other enzymes and peptides, such as aminopeptidase P, neutral endopeptidase, dipeptidyl peptidase-4, and kininase I, increases vascular permeability, which underlies this outcome.^15,16^

Thus, the search for new ACEi with fewer side effects is a crucial objective. Substituted isoindolines are of particular interest, as they have been shown to possess diverse biological activities, including serotonin uptake inhibition, antitumor, diuretic, and antihypertensive effects.^17,18^ In the cardiovascular system, for instance, the incorporation of an amidic functionality into the isoindolin-1-one and 5,6-dimethoxyisoindolin-1-one ring systems yielded highly cardioselective beta blockers.^19^ Also, Isoindolines targeting the ATP-binding pocket of Pyruvate Dehydrogenase Kinases 1–4 (PDK1–4), enzymes often upregulated in obesity, diabetes, and heart failure, have been shown to improve glucose tolerance and reduce hepatic steatosis, two key pathologies associated with obesity and type 2 diabetes.^20^ Moreover, Indoline derivatives have been tested for their antihypertensive potential, demonstrating promising results. Specifically, derivatives of 1-(3-mercaptopropanoyl)­indoline-2-carboxylic acid exhibit inhibitory activity against ACE that is three times greater than the activity produced by captopril.^21^ Then, the isoindoline derivatives have demonstrated potent activity with reduced toxicity and fewer side effects.^22–24^ The current contribution aimed to test a previously synthesized series of three isoindolines and five isoindoline-1,3-diones and evaluate them as nonpeptide ACEi for their potential as a new therapeutic alternative. The binding site was established with *in silico* (docking) studies, the kinetics of ACE inhibition with *in vitro* assays, and toxicity with *in vivo* experiments on mice. An analysis was conducted of how carbonyl groups influence enzyme inhibition.

A series of three isoindolines and five isoindoline-1,3-diones, nonpeptide derivatives, were synthesized following our previously described reports without any modification and under solventless conditions.^25–28^ Therefore, the characterization agrees with the reported values. The complete chemical characterization of these compounds is provided in the Supporting Information. However, a significant aspect of the present study involved the optimization of the assay used to examine ACE inhibition kinetics. Traditional methods described in the literature, typically performed in test tubes, are labor-intensive and inefficient for generating multiple kinetic curves concurrently. Thus, the technique was adapted to a 96-well plate to evaluate two compounds simultaneously and to accommodate up to 6 different concentrations of a compound per plate. Moreover, the method requires a minimum number of reagents and yields a final volume of 200 μL (Scheme 1 in the Supporting Information). Reagent and compound blanks were assessed separately to discard their absorbance. The development process was carried out using trinitrobenzenesulfonic acid (TNBS). The technique was validated by examining the IC_50_ of captopril, which was 3 nM (1.49 to 6.06 nM), consistent with the data reported in the literature.^29^ Subsequently, the synthesized compounds were tested at a maximum concentration of 1.5 mM (Table 1). A notable inhibition of the enzyme suggested potential pharmacological significance. The results and the chemical structures of the compounds are shown in Table 1.

**Table 1:**
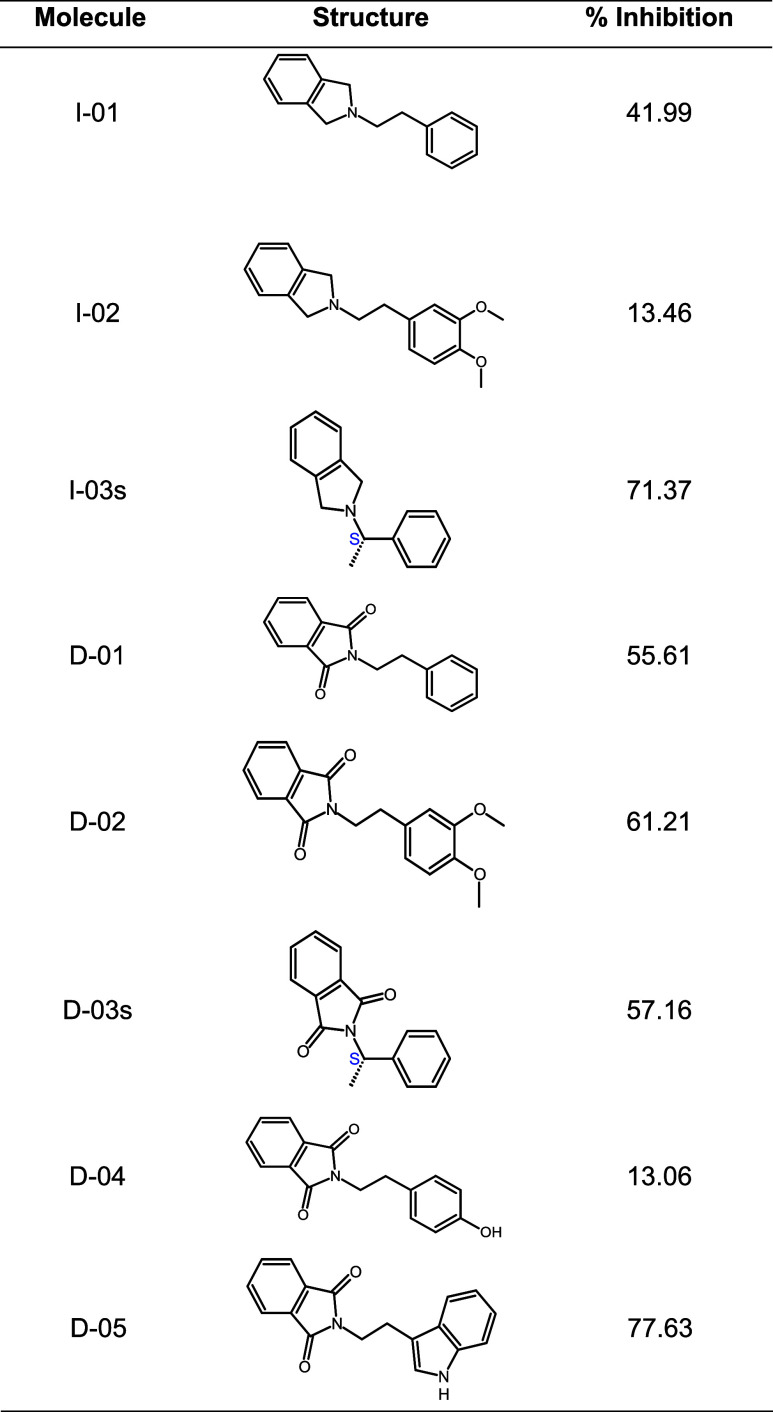
Structure of the Isoindolines and Isoindoline-1,3-diones, and the Results of *In Vitro* Screening as Possible Inhibitors of the Angiotensin-Converting Enzyme, Employing a 1.5 mM Concentration (n = 3) of Each Compound and HHL as the Substrate

Accordingly, at a concentration of 1.5 mM, I-01 and I-03s are the isoindoline derivatives that demonstrated the most significant inhibition of ACE (41.99 and 71.37%, respectively). In contrast, I-02 was eliminated from the study due to its low inhibition (13.46%). Among the isoindoline-1,3-dione derivatives, D-02 and D-05 displayed the best inhibition (61.21 and 77.63%, respectively). The IC_50_ was determined *in vitro* for the four most active compounds (I-01, I-03s, D-02, and D-05) and captopril (as a reference) (Table 2 and Figure 1).

**Table 2:** Inhibitory Concentration 50 (IC_50_) on the Angiotensin-Converting Enzyme from Rabbit Lung, Including Confidence Intervals of the Best Compounds Selected by the *In Vitro* Screening and the Reference Drug (Captopril)

**Compound**	**IC_50_ (μM)**	**Interval at 95%**
I-01	690.7	477.9 to 998.4
I-03s	543.6	481.7 to 613.4
D-02	586.0	367.7 to 934.1
D-05	416.4	334.5 to 518.4
Captopril	3.0 nM	1.49 to 6.06 nM
Captopril^30^		1.79 to 15.1 nM

**Figure 1:**
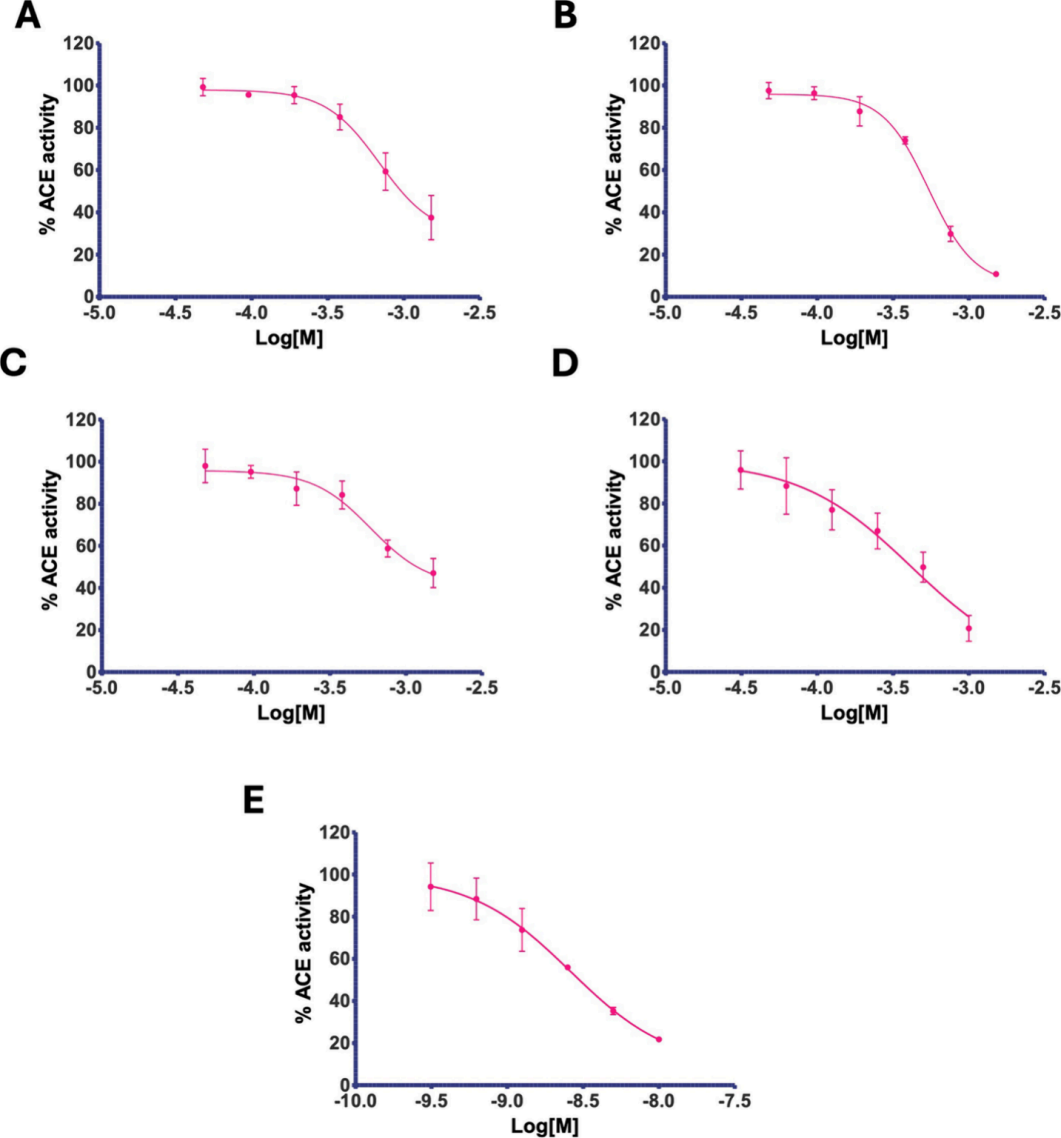
Plots of the inhibitory effect on the angiotensin-converting enzyme (ACE) from rabbit lung found in the *in vitro* assays with the selected compounds and the reference drug: **A**) **I-01**; **B**) **I-03s**; **C**) **D-02**; **D) D-05**; and **E)** captopril. The statistical analysis was carried out by nonlinear regression using the IC_50_ module (see Supporting Information document). The *X*-axis represents the logarithm of the molar concentration; the *Y*-axis depicts the percentage of ACE activity.

The value for captopril aligned with previously reported values in the literature, with overlap in the confidence intervals.^29^ Hence, the optimized technique reproduced the known drug’s reported value. The results for the isoindolines and isoindoline-1,3-diones showed no clear evidence of the action of the added carbonyl carbons in the latter molecules. Conversely, the presence of different substituents at the N position of isoindolines and isoindoline-1,3-diones appeared to influence inhibition. The tryptamine derivative (D-05) exhibited the lowest IC_50_ (416.4 μM) among all evaluated compounds. In this study, molecular docking was performed to gain insights into how isoindolines and isoindoline-1,3-diones inhibit ACE, and the results were compared with those of three reference drugs (captopril, enalapril, and lisinopril). Both families of test compounds displayed similar Gibbs’ free energy values and patterns for *K*_d_ and p*K*_d_, with an affinity for ACE comparable to that of captopril (Tables 3 and 4). Among the isoindoline-1,3-dione series, this molecule D-05 had the highest lipid/water partition coefficient (Table 6) and the most exergonic ΔG (Table 3). *In silico* analysis indicated it binds to the enzyme’s catalytic site (Table 4). Although D-05 did not demonstrate *in vitro* inhibitory capacity superior to captopril, it may perform better *in vivo* in future studies, since captopril’s peptidic nature reduces its bioavailability after biotransformation. Hence, the nonpeptidic D-05 may have greater bioavailability and, consequently, superior antihypertensive activity. Overall, isoindoline and isoindoline-1,3-dione derivatives showed similar ACE inhibition *in vitro*. The primary difference between the test compounds was their lethal dose 50 (LD_50_) value. Previous work from our group on phthalamide derivatives structurally related to isoindoline-1,3-diones showed that, although these compounds did not outperform captopril in *in vitro* ACE assays, they reduced blood pressure more effectively *in vivo*, with effects up to seven times greater than captopril in spontaneously hypertensive rats.^30^ This precedent supports the biological plausibility that the isoindoline-1,3-dione derivatives evaluated here may also exert antihypertensive effects. Future studies are needed to test these derivatives in spontaneously hypertensive rats to determine their blood pressure–lowering efficacy *in vivo*.

**Table 3:** Principal Parameters Obtained by Molecular Docking, Performed between the Test Compounds or Reference Drugs and the Human Angiotensin-Converting Enzyme

**Receptor**			
**Compound**	**Δ*G*** (kcal/mol)^a^	***K*_d_ (μM)**	**p*K*_d_**
I-01	–7.36	4.01	5.40
I-02	–8.63	0.469	6.33
I-03s	–7.73	2.16	5.67
D-01	–7.20	5.31	5.27
D-02	–7.90	1.62	5.79
D-03s	–7.12	6.02	5.22
D-04	–7.61	2.63	5.58
D-05	–8.09	1.18	5.93
**Captopril**	–7.120	6.010	5.22
**Enalapril**	–10.79	0.012	7.91
**Lisinopril**	–10.81	0.012	7.93

a Δ*G*, Gibbs free energy; *K*_d_, dissociation constant; (p*K*_d_) – log (10), dissociation constant.

**Table 4:** Amino Acid Residues Participating in the Interaction between the Human Angiotensin-Converting Enzyme and the Ligands: Phthalamides and the Reference Drugs

**Ligand**	**Residues**
**I-01**	Zn701, His353, Tyr523, His387, Tyr520, His513, Ser355, Glu384, Ala354, Phe457, His383, Ala356.
**I-02**	Met278, Trp279, Gly276, Ans277, Met169, Ala170, Glu376, Thr166, Asp377, Ala354, Gln369, Glu162, Cys370, Cys352.
**I-03s**	Zn701, His353, Tyr523, Glu384, His383, His513, Glu44, Glu411, Ala354.
**D-01**	Zn701, His353, Tyr523, His387, Tyr520, His513, Glu384, Ala354, Phe457, Glu411, His383, Ser355, Ala356.
**D-02**	Zn701, Tyr523, Tyr520, Phe457, His387, His383, His353, Ala354, Ser355, Glu384, Glu411, His513, Ala356.
**D-03s**	Zn701, His353, Tyr523, Glu384, His383, His513, His410, Phe391, Glu411, His387, Arg522, Ala356, Val518.
**D-04**	Zn701, Lys511, Tyr523, Tyr520, His387, His383, His353, Ser355, His513,, Gln281, Phe512.
**D-05**	Zn701, His353, Tyr523, Glu384, His383, Phe457, Tyr520, His513, Val518, Glu411, Ala354.
**Captopril**	Zn701, Lys511, Tyr523, Gly200, Glu384, Tyr520, His513, Gln281, Phe457.
**Enalapril**	Zn701, Lys511, His353, Tyr523, Glu384, Ala356, His387, Glu411, Val518, His513, Tyr520, Phe512, Phe457, Gln281.
**Lisinopril**	Zn701, Lys511, His353, Tyr523, His387, Glu384, Arg522, Glu411, His383, Ala354, Tyr520, His513, Phe512, Gln281.

Of the three isoindolines, I-02 showed the highest affinity for ACE. However, it exhibited little inhibition of the enzyme (13.46%) *in vitro* (Table 1). According to a more in-depth study (Tables 4 and 5) of the amino acid residues interacting with the ligand, as well as the types of interactions and binding distances, I-02 binds far from the enzyme’s catalytic site (Figure 2B), which partly explains its lack of significant inhibitory activity. This observation highlights a key methodological aspect of our *in silico* approach. In our docking protocol, the ligand was allowed to explore the entire enzyme surface rather than being restricted to a predefined catalytic site. This strategy not only estimates binding affinity (ΔG and p*K*_d_) but also identifies the specific binding site and the residues involved in the interaction. Such dual analysis helps determine whether the ligand engages the catalytic pocket or a potential allosteric region, providing a more realistic interpretation of the enzyme–inhibitor relationship. In the case of I-02, the high predicted affinity corresponds to binding at a noncatalytic site, explaining its low *in vitro* inhibition and demonstrating the interpretive value of our unrestricted docking approach. In the case of D-03, the stereogenic carbon is oriented toward Val518, while the carbonyl groups are directed toward Ala366 and Arg522, aligning the aromatic rings with the catalytic Zn^2+^ ion and generating a π-cation interaction that stabilizes the complex. Conversely, for I-03, the stereochemistry favors a molecular orientation that also enables π-cation interaction with the catalytic Zn^2+^ ion, positioning the compound appropriately within the active pocket. These spatial arrangements of both molecules highlight the important role of configuration in their interaction with ACE, as the proper alignment of the carbonyl and aromatic moieties promotes Zn^2+^ coordination and hydrogen bonding—features widely recognized as critical determinants of inhibitory potency. On the other hand, I-01 and I-03s have affinity for ACE comparable to that of I-02 (p*K*_d_), and both bind to the catalytic site of the enzyme (Figure 2A and Figure 2C). Additionally, they have interactions with important amino acid residues at the catalytic site, like those involved in the binding of the reference drugs. Such residues encompass His513, Tyr523, Glu384, and the Zn atom (Table 4). The family of isoindolines displays predominantly π interactions.

**Table 5:** Main Interactions and Binding Distances Corresponding to the Interaction between the Isoindolines/Isoindoline-1,3-dione and the Angiotensin-Converting Enzyme

**Ligand**	**Interactions**
**I-01**	Electrostatic: Ala354 to 2.78 Å, Glu384 to 1.88 Å, Glu411 to 4.93 Å
	π-π: Tyr523 to 3.80 Å
	π-donor: Ala356 to 4.09 Å
	π-cation: Zn to 3.1 Å
**I-02**	Electrostatic: Glu376 to 5.16 Å, Asp377 to 2.01 Å, Glu162 to 3.74 Å
	π-donor: Asn277 to 3.76 Å, Thr166 to 3.45 Å
	π-anion: Asp377 to 4.15 Å, Glu162 to 3.33 Å
	hydrophobic: Ala354 to 4.55 Å, Thr166 to 3.38 Å
**I-03s**	Electrostatic: Glu354 to 2.73 Å, Glu411 to 5.13 Å
	π-π: Tyr523 to 4.34 Å
	π-cation: Zn to 2.20 Å, His513 to 5.51 Å, His353 to 4.35 Å
	hydrophobic: Val518 to 5.29 Å
	H-bond: Ala354 to 2.73 Å
**D-01**	π-π: His387 to 4.36 Å, His383 to 5.61 Å, Tyr523 to 4.06 Å
	π-cation: Zn to 3.08 Å
	hydrophobic: Glu384 to 2.79 Å
	H-bond: Tyr523 to 2.80 Å, His513 to 2.06 Å, His553 to 2.26 Å
**D-02**	π-π: Tyr523 to 4.13 Å, His387 to 4.29 Å, His383 to 2.95 Å
	π-cation: Zn to 4.68 Å
	hydrophobic: His387 to 3.23 Å, Glu384 to 2.80 Å
	H-bond: Tyr523 to 2.60 Å, His513 to 1.99 Å, His353 to 2.43 Å, Ala356 to 1.71 Å
**D-03s**	π-π: His387 to 3.95 Å
	π-cation: Zn to 3.99 Å
	π-anion: Glu411 to 4.93 Å
	hydrophobic: Val518 to 3.88 Å
	H-bond: Ala356 to 1.95 Å, Tyr523 to 3.05 Å, Arg522 to 2.37 Å
**D-04**	π-π: Tyr523 to 4.98 Å, His513 to 4.84 Å
	π-cation: Zn to 3.08 Å, His513 to 4.09 Å
	hydrophobic: Val518 to 4.98 Å
	H-bond: Lys511 to 1.82 Å, His353 to 1.68 Å, His513 to 2.13 Å
**D-05**	π-π: Tyr523 to 3.99 Å and 4.65 Å
	π-donor: Tyr523 to 2.24 Å
	π-cation: Zn to 4.78 Å, His513 to 4.75 Å
	hydrophobic: Val518 to 4.37 Å
	H-bond: His513 to 2.17 Å, His353 to 2.09 Å, Tyr523 to 3.04 Å, Glu411 to 2.17 Å

**Figure 2:**
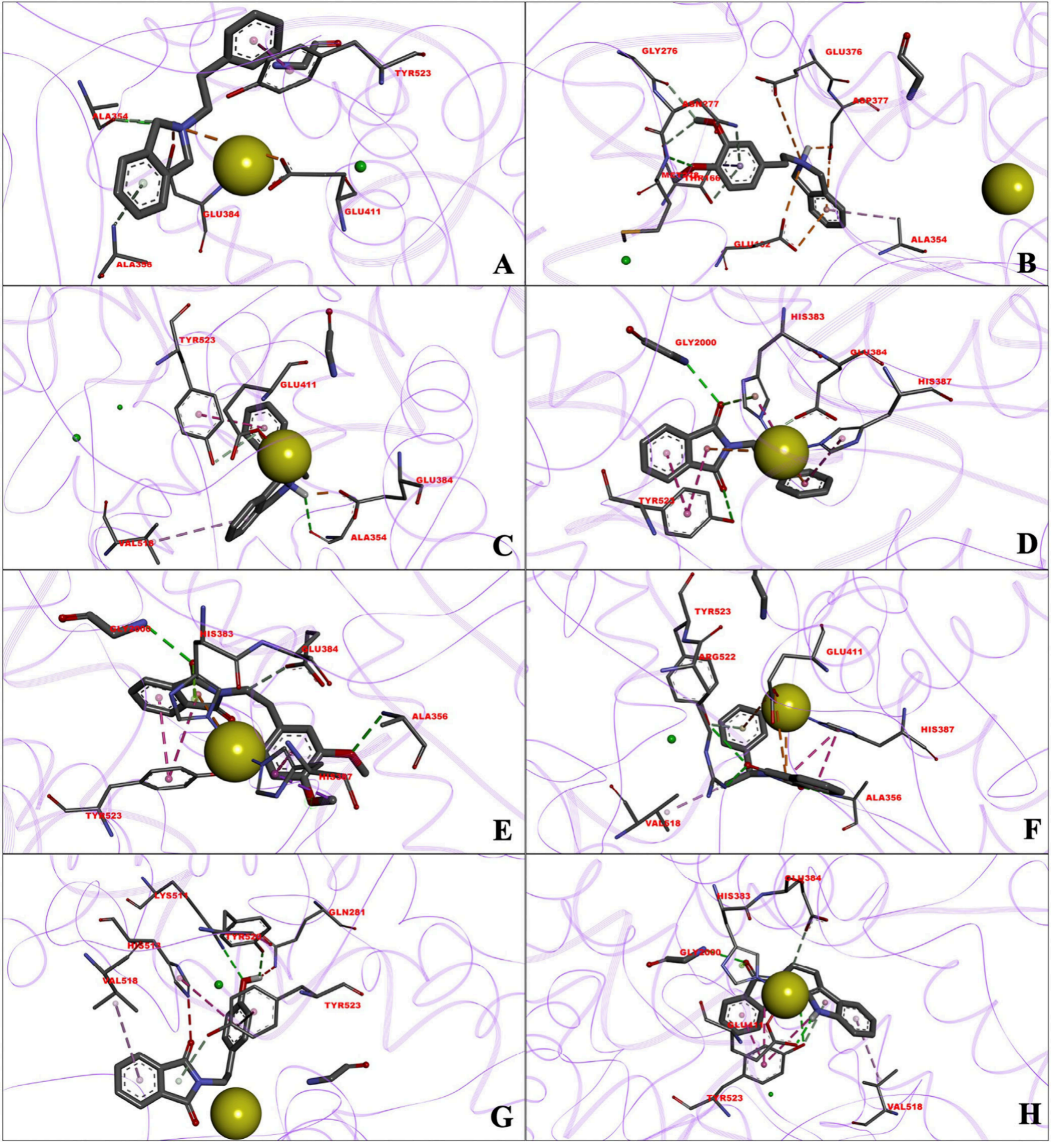
Principal amino acid residues involved in the interaction between the test compounds and the human angiotensin-converting enzyme, obtained by molecular docking. The zinc cation (yellow) shows the catalytic site of the enzyme. Binding site of A) I-01, B) I-02, C) I-03s, D) D-01, E) D-02, F) D-03s, G) D-04, and H) D-05.

Among the five isoindoline-1,3-diones, there was a similar affinity for ACE. The highest affinity and the most significant inhibitory activity were observed for D-02 and D-05 (Tables 1 and 2). Based on an in-depth analysis, this entire family binds at the catalytic site of the enzyme (Figure 2. D-H) and interacts with key amino acid residues (Table 4). Isoindoline-1,3-diones mainly form π and hydrogen-bond interactions. These stabilize the enzyme–inhibitor complex (Table 5) and are mediated principally by the addition of the two carbonyl carbons in positions 1 and 3. For instance, the primary difference between I-02 and D-02 in relation to the inhibitory effect (being 13.46% and 61.21%, respectively) is the presence of the carbonyl carbons in the latter. Compared to I-02, the interactions are more favorable for D-02 (Table 5), which binds to the catalytic site (Figure 2 and Table 4). Other compounds containing two carbonyl carbons showed a positive trend, although the pattern was not observed for all the ligands currently evaluated. This trend suggests that the incorporation of two carbonyl groups stabilizes the enzyme–inhibitor complex, particularly in the isoindoline-1,3-dione derivatives, which exhibit more favorable interactions within the catalytic site. The additional carbonyl groups likely enhance electronic delocalization and facilitate hydrogen bonding with key residues, improving overall binding geometry. Nevertheless, this effect was not uniformly observed across all analogs, indicating that other structural elements—especially the type and position of N-substituents—also modulate affinity and orientation. These observations are consistent with the *in silico* docking results, which reveal a partial but significant trend linking the carbonyl functionality to increased binding. This suggests further SAR investigations to clarify its specific role, and a larger set of both families is required to establish a more accurate and conclusive pattern.

Additionally, acute toxicity was assessed for the eight synthesized compounds (Table 6). The LD_50_ was greater than 1600 mg/kg for all isoindoline-1,3-diones and less than 1000 mg/kg for the isoindolines. The lower toxicity of the former may be due to their liposolubility and to the secondary metabolites they generate. These two families of compounds are closely related, differing only in the presence of two carbonyl groups in the isoindoline-1,3-dione structure.

**Table 6:**
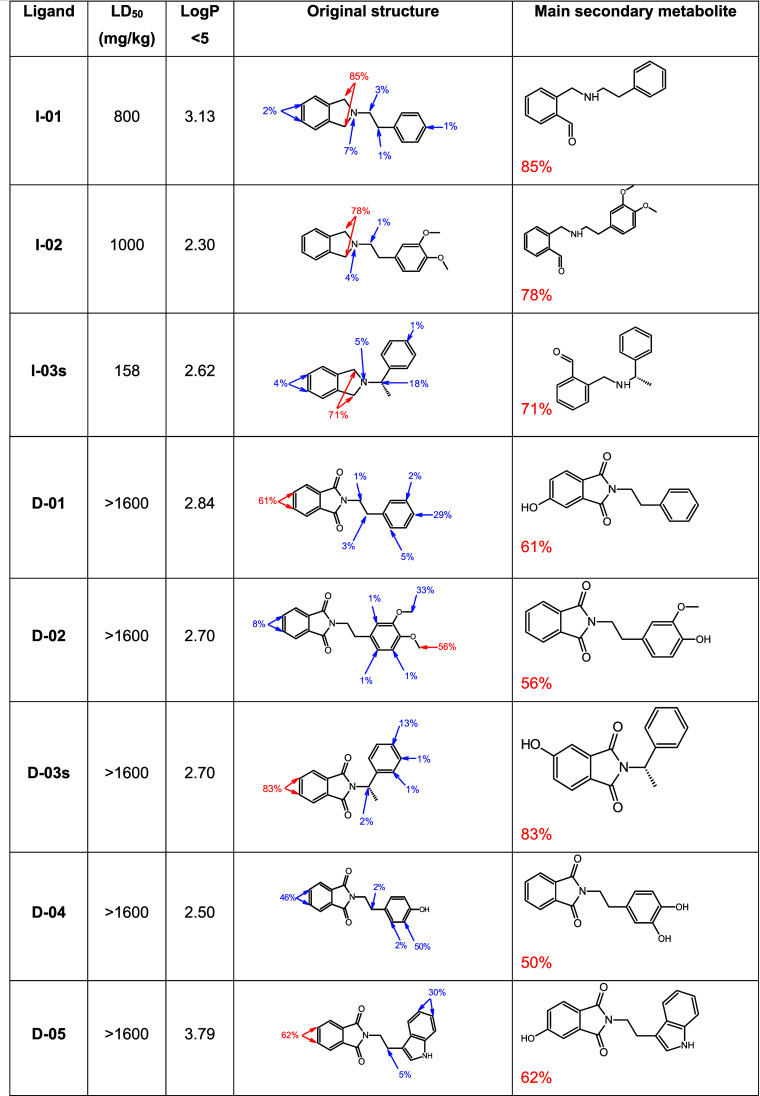
Evaluation of the Metabolism of Isoindolines and Isoindoline-1,3-diones by the 3A4 Isoform of CYP450, Carried out by StarDrop Software Using the P450 Module^a^

a The third column presents the probabilistic regions of the metabolism of the molecule by cytochrome.

An *in silico* study was conducted to predict metabolites induced by the CYP3A4 isoform in the two families of compounds. It was decided to use the 3A4 isoform of CYP450 because it is responsible for the biotransformation of most drugs. As a result of bearing the carbonyl carbons in positions 1 and 3, isoindoline-1,3-diones are prevented from being biotransformed in these positions. Whereas the main secondary metabolites generated in the isoindolines are aldehyde derivatives, those produced by isoindoline-1,3-diones are oxidized derivatives of alcohols (Table 6). Compared to alcohol derivatives, aldehyde derivatives tend to be more reactive with other biomolecules. Besides favoring an increase in the LD_50_, the addition of the two carbonyl carbon groups decreased the lipid/water coefficient, making the isoindoline-1,3-diones more hydrosoluble. The lipid/water coefficient of these compounds is within the acceptable range of reference values established by Lipinski.^31^ Interestingly, the presence of two carbonyl carbons also correlates with reduced toxicity in the *in vivo* LD_50_ assays, where all isoindoline-1,3-dione derivatives exhibited values above 1600 mg/kg. This observation aligns with the *in silico* metabolism predictions, which indicated that isoindoline-1,3-diones generate less reactive alcohol-type metabolites compared to the aldehyde derivatives formed from isoindolines. These results support the idea of a potential protective role of the carbonyl groups, though a more comprehensive evaluation of both structural families is needed to fully confirm this relationship.

In summary, a worthwhile strategy for discovering better treatments for HTN is the design of new nonpeptide ACE inhibitors. Two families of such inhibitors, isoindolines and isoindoline-1,3-diones, were herein synthesized, thoroughly analyzed *in silico* for their binding to ACE, and evaluated *in vitro* for their inhibition of the same enzyme. Using an optimized ACE kinetics assay, D-05 showed the best *in vitro* ACE inhibition (IC_50_ = 416.4 μM). Additionally, it exhibited low toxicity (LD_50_ > 1600 mg/kg) and generated an alcohol derivative as the main secondary metabolite. *In silico* studies predict that D-05 binds to ACE at the catalytic site, where it interacts with the principal amino acid residues involved in captopril binding. Although the *in silico* docking analysis did not show a definitive correlation between carbonyl carbons and increased ACE inhibition, the *in vivo* LD_50_ data and metabolic predictions indicate a clear connection between these groups and reduced toxicity. Finally, a potential nonpeptidic ACE inhibitor, D-05, has been identified. Future studies will be required to determine whether this compound can effectively reduce blood pressure in hypertensive *in vivo* models.

## Supplementary Material



## Notes

Full experimental details for the characterization of isoindolines and isoindoline-1,3-diones, preparation of solutions, *in vitro* ACE inhibition assays, *in vivo* acute toxicity evaluation, molecular docking studies, and statistical analysis (PDF)

## Abbreviations

ACEi: angiotensin-converting enzyme inhibitors
HTN: hypertension
RAS: renin-angiotensin system
PDK1–4: Pyruvate Dehydrogenase Kinases 1–4
TNBS: trinitrobenzenesulfonic acid
HHL: N-Hippuryl-His-Leu
