# Supplementary material for: Isoindolines and Isoindoline-1,3-diones as Nonpeptide ACE Inhibitors: An In Silico and In Vitro Modeling Approach
Source: ACS Med Chem Lett. 2026 Feb 9;17(3):563–70. doi: 10.1021/acsmedchemlett.5c00507 (PMC12989995; doi:10.1021/acsmedchemlett.5c00507)
Supplement: Supplementary file 1 [file ml5c00507_si_001.pdf]

Supporting information for

Isoindolines and isoindoline-1,3-diones as non-peptide ACE inhibitors: An *in silico* and *in vitro* modeling approach.

Jessica E. Rodríguez [a], Jesús A. Lagos-Cruz [b], Rafael Villalobos-Molina [c], Roberto I. Cuevas-Hernández [b], Itzell A. Gallardo-Ortíz *[c], and Erik Andrade-Jorge*[b]

[a]Área de Química Computacional y Modelado Molecular, Bioquímica Clínica, Carrera de Químico Farmacéutico Biólogo, Facultad de Estudios Superiores Zaragoza. Universidad Nacional Autónoma de México. Av. Guelatao con Av. Exploradores, Ejército de Oriente, Iztapalapa, 09320, Ciudad de México, México.
[b]Laboratorio de Investigación en Biomedicina y Toxicología, Sección de Estudios de Posgrado e Investigación. Escuela Superior de Medicina del Instituto Politécnico Nacional, Plan de San Luis y Díaz Mirón s/n Casco de Santo Tomás, 11340, Mexico City, México.
[c]Unidad de Investigación en Biomedicina y Carrera de Enfermería, Facultad de Estudios Superiores-Iztacala, Universidad Nacional Autónoma de México. Av. de los Barrios 1, Los Reyes Iztacala, Tlalnepantla, 54090, Estado de México, México.
(*Corresponding authors)
E-mail addresses: eandrade@ipn.mx (EAJ), igallardo@comunidad.unam.mx (IAGO)

1. Experimental sections

1.1 Synthesis and characterization

The compounds were synthesized as previously reported by our group [1–4]. Briefly, all reactions were performed under solventless conditions in 50 mL oven-dried flasks at the melting point of the reagents, using 1.1 mmol of α,α-dibromo-o-xylene, or phthalic anhydride to obtain the isoindolines or isoindoline-1,3-diones, respectively, and 1 mmol of the respective amine, under constant stirring with a stirring bar for 10-15 min. The reaction was cooled to room temperature and monitored by thin-layer chromatography. The reaction mixture was purified with the appropriate solvent, ethyl acetate, and water (pH 10). The mixture was sonicated until a white powder formed, and then it was concentrated using a standard rotary evaporator. The reagents and solvents were used as received from the commercial supplier, Sigma-Aldrich (St. Louis, MO, USA). Melting points were read on a Stuart® SMP40 automatic melting point apparatus and were uncorrected. Chemical characterization was performed using a 100 FT-IR spectrometer (PerkinElmer) equipped with a universal ATR accessory and a Varian Mercury 300 (^{1}H, 300 MHz; ^{13}C, 75 MHz) spectrometer, with tetramethylsilane (TMS) as the internal reference.

Electrospray ionization (ESI) high-resolution mass spectrometry was performed on a Bruker micrOTOf-Q-II instrument. The parameters reported are chemical shifts in ppm (δ), the integration area, multiplicity (s=singlet, d=doublet, t=triplet, q=quartet, and m=multiplet), and coupling constants (Hz).

Characterization of 2-phenethylisoindoline hydrobromide (I-01)

White solid; yield 90%; mp 165–166 °C. **^{1}H NMR** (300 MHz, DMSO-d_6) δ 7.36 (*m*, H-4,5,6,7,13,14,15,16,17), 4.72 (*dd*, H-1,3), 3.65 (*t*, H-10), 2.85 (*t*, H-11). **^{13}C NMR** (75 MHz, $CDCl_3$) δ 137.9 (C-8,9), 134.9 (C-12), 129.2 (C-13,14,16,17), 127.5 (C-15), 127.4 (C-5,6), 123.5 (C-4,7), 58.4 (C-1,3), 55.4 (C-10), 33.5 (C-11). **IR (ATR, cm^{-1})** ν_{max}: 3031 (C-H, Aromatic), 2908 (C-H, Aliphatic), 2546 (NH^+), 1705 (C=O), 1600 (C=C), 1427 (CH_2), 1391 (C-N). **MS (*m/z*)**: 224.1447 (M+1).

Characterization of 2-(3,4-dimethoxyphenethyl)isoindoline hydrobromide (I-02)

White solid, yield 80%; mp 197-198 °C; **^{1}H NMR** (300 MHz, DMSO-d_6) δ 7.40 (*m*, H-4,5,6,7), 6.95 (*m*, H-13,16,17), 4.71 (*s*, H-1,3), 3.74 (*s*, H-19), 3.71 (*s*, H-18), 3.64 (*t*, H-10), 3.00 (*t*, H-11); **^{13}C NMR** (75 MHz, $CDCl_3$) δ 149.4 (C-15), 148.4 (C-14), 134.9 (C-8,9), 129.8 (C-12), 129.1 (C-4,7), 123.5 (C-5,6), 121.3 (C-17), 113.2 (C-16), 112.6 (C-13), 58.5 (C-1,3), 56.2 (C-18,19), 55.7 (C-10), 31.8 (C-11). **IR (ATR, cm^{-1})** ν_{max}: 3063 (C-H, Aromatic), 2990 (C-H, Aliphatic), 2842 (O-CH_3, Aliphatic), 2547 (NH^+), 1600 (C=C), 1462 (CH_2), 1453 (CH_3), 1390 (C-N), 1228 (O-CH_3). **MS (*m/z*)**: 284.1624 (M+1).

Characterization of (*S*)-2-(1-phenylethyl)isoindoline hydrobromide (I-03s)

White solid, yield 35%; mp 210-211 °C; **^{1}H NMR** (750 MHz, DMSO-d_6) δ 7.15-7.85 (*m*, H-4-7, 13-17), 4.6 (*q*, H-10), 4.28 (*dd*, H-1,3), 1.90 (*d*, H-11), **^{13}C NMR** (189 MHz DMSO-d_6) δ 136.1 (C-12), 133.2 (C-8,9), 130.0 (C-14,16), 129.8 (C-13,17), 129.2 (C-15), 128.7 (C-5,6), 123.1 (C-4,7), 67.8 (C-10), 58.1 (C-1,3), 19.6 (C-11). **IR (ATR, cm^{-1})** ν_{max}: 3014 (C-H, Aromatic), 2978 (C-H, Aliphatic), 2505 (NH^+), 1584 (C=C), 1458 (CH_2), 1444 (CH_3), 1409 (C-N). **MS (*m/z*)**: 224.1359 (M+1).

Characterization of 2-phenethylisoindoline-1,3-dione (D-01)

White solid; yield 85%; mp 130–131 °C. **^{1}H NMR** (300 MHz, $CDCl_3$) δ *7.82 (dd, J = 5.5, 3.0 Hz, 2H, H-4,7), 7.70 (dd, J = 5.6, 3.0 Hz, 2H, H-5,6), 7.32–7.18 (m, 5H, H-13,14,15,16,17), 3.92 (t, J = 7.4 Hz, 2H, H-10), 2.98 (t, J = 7.4 Hz, 2H, H-11).* ***^{13}C NMR*** *(75 MHz, $CDCl_3$)* δ *168.1 (C-1,3), 137.9 (C-12), 133.9 (C-5,6), 132.0 (C-8,9), 128.8 (C-14,16), 128.5 (C-13,17), 126.6 (C-15), 123.2 (C-4,7), 39.2 (C-10), 34.6 (C-11).* **IR (ATR, cm^{-1})** ν_{max}: 3031 (C–H, Aromatic), 2935 (C–H, Aliphatic), 1705 (C=O), 1600 (C=C), 1427 (CH_2), 1391 (C–N). **HRMS (*m/z*)**: 274.0752 (M+Na), calculated for $C_{16}H_{13}NO_2Na$: 274.0838.

Characterization of 2-(3,4-dimethoxyphenethyl)isoindoline-1,3-dione (D-02)

Light yellow solid; yield 90%; mp 171–172 °C. **^{1}H NMR** (300 MHz, $CDCl_3$) δ *7.81 (dd, J = 5.5, 3.0 Hz, 2H, H-4,7), 7.70 (dd, J = 5.4, 3.1 Hz, 2H, H-5,6), 6.78–6.73 (m, 3H, H-13,16,17), 3.90 (t, J = 7.5 Hz, 2H, H-10), 3.83 (s, 3H, H-18), 3.80 (s, 3H, H-19), 2.93 (t, J = 7.5 Hz, 2H, H-11).* ***^{13}C NMR*** *(75 MHz, $CDCl_3$)* δ *168.2 (C-1,3), 148.7 (C-14), 147.6 (C-15), 133.9 (C-12), 132.0 (C-5,6), 130.4 (C-8,9), 123.1 (C-4,7), 120.8 (C-17), 111.8 (C-16), 111.1 (C-13), 55.7 (C-18,19), 39.3 (C-10), 34.0 (C-11).* **IR (ATR, cm^{-1})** ν_{max}: 3063 (C–H, Aromatic), 2943 (C–H, Aliphatic), 2842 (O–CH_3, Aliphatic), 1705 (C=O), 1600 (C=C), 1466 (CH_2), 1427 (CH_3), 1394 (C–N), 1228 (O–CH_3). **HRMS (*m/z*)**: 334.0956 (M+Na), calculated for $C_{18}H_{17}NO_4Na$: 334.1049.

Characterization of (*S*)-2-(1-phenylethyl)isoindoline-1,3-dione (D-03s)

Yellow oil; yield 89%. **^{1}H NMR** (300 MHz, $CDCl_3$) *δ 7.80 (dd, J = 5.6, 2.9 Hz, 2H, H-4,7), 7.68 (dd, J = 5.6, 2.9 Hz, 2H, H-5,6), 7.52–7.50 (m, 2H, H-14,16), 7.49–7.48 (m, 2H, H-13,17), 7.35–7.22 (m, 1H, H-15), 5.56 (q, J = 7.3 Hz, 1H, H-10), 1.93 (d, J = 7.3 Hz, 3H, H-11).* ***^{13}C NMR*** *(75 MHz, $CDCl_3$) δ 168.1 (C-1,3), 140.2 (C-12), 133.8 (C-5,6), 131.9 (C-8,9), 128.4 (C-15), 127.6 (C-14,16), 127.4 (C-13,17), 123.1 (C-4,7), 49.5 (C-10), 17.5 (C-11).* **IR (ATR, cm^{-1})** ν_{max}: 3063 (C–H, aromatic), 2935 (C–H, aliphatic), 1703 (C=O), 1600 (C=C), 1427 (CH_2), 1425 (CH_3), 1384 (C–N). **HRMS (*m/z*)**: 274.0774 (M+ Na), calculated for $C_{16}H_{13}NO_2Na$: 274.0838.

Characterization of 2-(4-hydroxyphenethyl)isoindoline-1,3-dione (D-04)

White solid; yield 89%; mp 237–239 °C. ^{1}H NMR (750 MHz, DMSO-d_6) δ 9.18 (*s*, 1H, OH-15), 7.83–7.80 (*m*, 4H, H-4,5,6,7), 6.96 (*d*, *J* = 8.4 Hz, 2H, H-13,17), 6.63 (*d*, *J* = 8.4 Hz, 2H, H-14,16), 3.73 (*t*, *J* = 7.4 Hz, 2H, H-10), 2.79 (*t*, *J* = 7.4 Hz, 2H, H-11). **^{13}C NMR** (189 MHz, DMSO-d_6) δ 168.2 (C-1,3), 156.0 (C-15), 134.8 (C-5,6), 131.9 (C-8,9), 130.0 (C-12), 128.6 (C-13,17), 123.6 (C-4,7), 115.6 (C-14,16), 39.5 (C-10), 33.2 (C-11). **IR (ATR, cm^{-1})** ν_{max}: 3255 (O-H) 3026 (C-H, aromatic), 2955 (C-H, aliphatic), 1703 (C=O), 1613 (C=C), 1466 (CH_2), 1384 (C-N). **HRMS (*m/z*)**: 290.0715 (M+Na), calculated for $C_{16}H_{13}NO_3Na$: 290.0787.

Characterization of 2-(2-(1*H*-indol-3-yl)ethyl)isoindoline-1,3-dione (D-05)

Light yellow solid; yield 80%; mp 182–184 °C. **^{1}H NMR** (750 MHz, DMSO-d_6) δ 10.83 (*s*, 1H, NH-14), 7.85 (*dd*, *J* = 5.5, 3.0 Hz, 2H, H-7,4), 7.81 (*dd*, *J* = 5.2, 3.1 Hz, 2H, H-5,6), 7.55 (*d*, *J* = 7.9 Hz, 1H, H-15), 7.32 (*d*, *J* = 8.1 Hz, 1H, H-18), 7.17 (*s*, 1H, H-13), 7.06 (*t*, *J* = 7.5 Hz, 1H, H-16), 6.97 (*t*, *J* = 7.4 Hz, 1H, H-17), 3.85 (*t*, *J* = 7.5 Hz, 2H, H-10), 3.03 (*t*, *J* = 7.7 Hz, 2H, H-11). **^{13}C NMR** (189 MHz, DMSO-d_6) δ 168.2 (C-1,3), 136.7 (C-20), 134.8 (C-5,6), 132.0 (C-8,9), 127.5 (C-19), 123.4 (C-4,7/13), 121.4 (C-16), 118.8 (C-17), 118.4 (C-18), 111.9 (C-12), 111.0 (C-15), 38.7 (C-10), 24.3 (C-11). **IR (ATR, cm^{-1})** ν_{max}: 3374 (N-H) 3031 (C-H, aromatic), 2986 (C-H, aliphatic), 1703 (C=O), 1611 (C=C), 1466 (CH_2), 1384 (C-N). **HRMS (*m/z*)**: 313.0869 (M+Na), calculated for $C_{18}H_{14}N_2O_2Na$: 313.0947.

The compounds were handled, stored, and evaluated in accordance with established chemical and analytical standards to ensure their suitability for biological testing.

1.2. Solutions

A phosphate buffer solution (0.1 M, pH 8.3) was prepared by dissolving K_2HPO_4 (8.07 g) and KH_2PO_4 (0.5 g) in a volumetric flask. NaCl (8.76 g) and Na_2SO_4 (28.4 g) were added to achieve concentrations of 0.3 M and 0.4 M, respectively, and the volume was adjusted to 500 mL with distilled water. The ACE stock solution (0.05

U/mL) was prepared in phosphate buffer using lyophilized ACE powder from rabbit lung (Sigma-Aldrich, cat. no. A6778-1UN) and stored at −70 °C. The substrate *N*-Hippuryl-His-Leu (HHL, Sigma-Aldrich, cat. no. H1635) was dissolved in phosphate buffer to obtain a 10 mM solution. The TNBS solution (Sigma-Aldrich, cat. no. P2297-10ML; 3.518 mL) was diluted with distilled water to a final volume of 40 mL (15 mM). All solutions were mixed using an Analog Vortex Mixer (Thomas Scientific).

1.3. Angiotensin-converting enzyme activity assay

Standard and inhibitory curves were constructed by adding 20 μL of each solution (HHL, inhibitor, and ACE) to a 96-well microplate (Scheme 1). The microplate was incubated for 6 h at 37 °C in a water bath (Felisa). The reaction was terminated by adding 20 μL of TNBS solution to each well. After incubation, in the dark, at room temperature for 20 min, absorbance was measured at 415 nm using a microplate reader (Epoch I Biotek Gene 5 v 3.11) with 60 s of premixing. Assays were performed in triplicate, and data are expressed as the average of three values. IC_{50} values were determined by nonlinear regression analysis.

1.4. Acute Toxicity (LD_{50}) in Mice

The LD_{50} was estimated using Lorke's method. Briefly, male CD-1 mice (20–25 g) were divided into three groups (n = 3 per group) for the first phase. A single dose (10, 100, or 1000 mg/kg) of the test compound (isoindoline or isoindoline-1,3-dione) was administered via intraperitoneal injection. Animals were observed for 24 h to record mortality. Subsequently, three new groups were established to administer higher doses (1200, 1400, and 1600 mg/kg for isoindoline-1,3-diones; 250, 500, and 750 mg/kg for isoindolines), and mortality was monitored for another 24 h. Results were analyzed using the Reed-Muench method [5,6]. All procedures were approved by the institutional Bioethics Committee (protocol No. 1497) and adhered to the Mexican Official Norm for the production, care, and use of laboratory animals (NOM-062-ZOO-1999, SAGARPA, Mexico) and the NIH Guide for the Care and Use of Laboratory Animals.

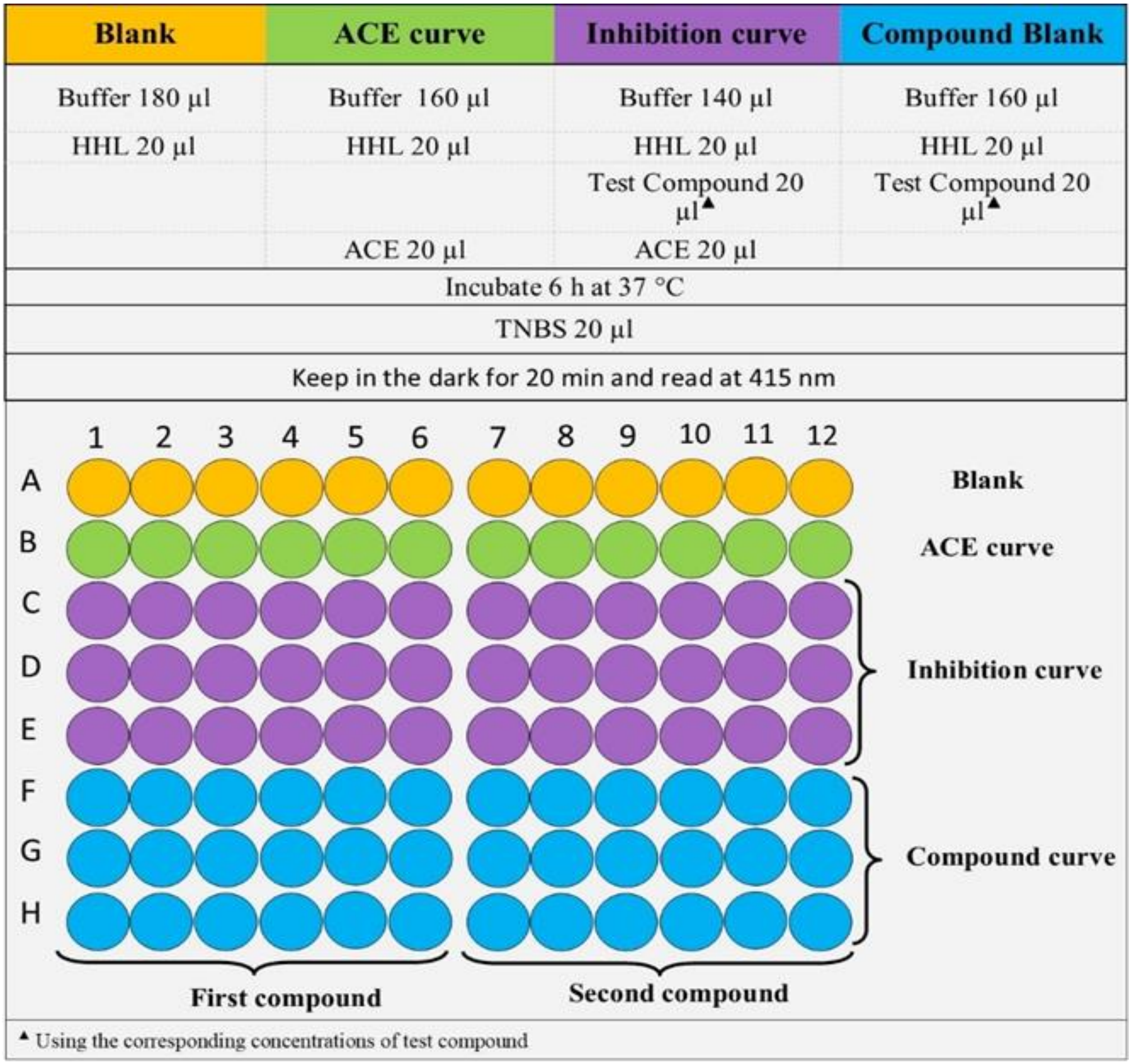

Scheme 1. Representation of the pattern of compounds in a 96-well microplate, used for the improved method of determining the inhibition of the angiotensin-converting enzyme (ACE). Using the illustrated pattern, different curves were constructed to evaluate the inhibitory activity of isoindolines and isoindoline-1,3-diones on ACE.

1.5. Theoretical calculations

Metabolite prediction (mediated by the CYP450 3A4 isoform) and calculation of the lipid/water partition coefficient (log *P*) were performed using the P450 module of StarDrop software.

1.6. Docking

Ligand conformational analysis was performed using the PM6 semi-empirical method in GaussView 5.0.9 and Gaussian 09 software [7]. Physiological conditions (pH 7.4) were assumed for protonation states. A hybrid Lamarckian genetic algorithm [8], an initial population of 100 randomly placed individuals, Kollman partial charges for protein atoms, and Gasteiger charges for ligands were implemented using AutoDock Tools 1.5.4 [8] and Raccoon software [9]. The crystal structure of human ACE was retrieved from the Protein Data Bank (PDB code: 1O86) [10]. The grid center was defined at X = 37.531, Y = 33.432, and Z = 44.336, with a grid box size of 80 × 60 × 70 Å and 0.375 Å spacing. Docking was performed using AutoDock4 on a Linux operating system (Fedora 22) to determine the Gibbs free energy (ΔG), dissociation constant (K_d), pK_d (-logK_d), number of interactions, binding distance, and binding type.

1.7. Statistical analysis

Data from the ACE inhibitory assay are expressed as mean values with 95% confidence intervals. IC_{50} values were calculated by nonlinear regression analysis using the “log(inhibitor) vs. response” model in GraphPad Prism software (version 5.03, GraphPad Software Inc., San Diego, CA, USA).

References

(1)Andrade-Jorge, E.; Bahena-Herrera, J. R.; Garcia-Gamez, J.; Padilla-Martínez, I. I.; Trujillo-Ferrara, J. G. Novel Synthesis of Isoindoline/Isoindoline-1,3-Dione Derivatives under Solventless Conditions and Evaluation with the Human D2 Receptor. *Medicinal Chemistry Research* **2017**, *26* (10), 2420–2431. https://doi.org/10.1007/S00044-017-1942-6.

(2)Andrade-Jorge, E.; Sánchez-Labastida, L. A.; Soriano-Ursúa, M. A.; Guevara-Salazar, J. A.; Trujillo-Ferrara, J. G. Isoindolines/Isoindoline-1,3-Diones as AChE Inhibitors against Alzheimer's Disease, Evaluated by an Improved Ultra-Micro Assay. *Medicinal Chemistry Research* **2018**, *27* (9), 2187–2198. https://doi.org/10.1007/S00044-018-2226-5.

(3)Ruiz-Maciel, O.; Padilla-Martínez, I. I.; Sánchez-Labastida, L. A.; Soriano-Ursúa, M. A.; Andrade-Jorge, E.; Trujillo-Ferrara, J. G. Inhibitory Activity on Cholinesterases Produced by Aryl-Phthalimide Derivatives: Green Synthesis, in Silico and in Vitro Evaluation. *Medicinal Chemistry Research* **2009**, *29* (6), 1030–1040. https://doi.org/10.1007/S00044-020-02543-2.

(4)Andrade-Jorge, E.; Bribiesca-Carlos, J.; Martínez-Martínez, F. J.; Soriano-Ursúa, M. A.; Padilla-Martínez, I. I.; Trujillo-Ferrara, J. G. Crystal Structure, DFT Calculations and Evaluation of 2-(2-(3,4-Dimethoxyphenyl) Ethyl)Isoindoline-1,3-Dione as AChE Inhibitor. *Chem Cent J* **2018**, *12* (1), 74. https://doi.org/10.1186/S13065-018-0442-1.

(5)Lorke, D. A New Approach to Practical Acute Toxicity Testing. *Arch Toxicol* **1983**, *54* (4), 275–287. https://doi.org/10.1007/BF01234480.

(6)Chinedu, E.; Arome, D.; Ameh, F. S. A New Method for Determining Acute Toxicity in Animal Models. *Toxicol Int* **2013**, *20* (3), 224–226. https://doi.org/10.4103/0971-6580.121674.

(7)*Gaussian ~09 Revision D.01 – ScienceOpen*. https://www.scienceopen.com/document?vid=839f33cc-9114-4a55-8f1a-3f1520324ef5 (accessed 2022-09-26).

(8)Morris, G. M.; Goodsell, D. S.; Halliday, R. S.; Huey, R.; Hart, W. E.; Belew, R. K.; Olson, A. J. Automated Docking Using a Lamarckian Genetic Algorithm and an

Empirical Binding Free Energy Function. *J Comput Chem* **1639**, *19* (14), 16391662. https://doi.org/10.1002/(SICI)1096-987X(19981115)19:14.

(9)Forli, S.; Huey, R.; Pique, M. E.; Sanner, M. F.; Goodsell, D. S.; Olson, A. J. Computational Protein–Ligand Docking and Virtual Drug Screening with the AutoDock Suite. *Nature Protocols 2016 11:5* **2016**, *11* (5), 905–919. https://doi.org/10.1038/nprot.2016.051.

(10)Natesh, R.; Schwager, S. L. U.; Sturrock, E. D.; Acharya, K. R. Crystal Structure of the Human Angiotensin-Converting Enzyme–Lisinopril Complex. *Nature 2003 421:6922* **2003**, *421* (6922), 551–554. https://doi.org/10.1038/nature01370.
